# Ameliorating of Memory Impairment and Apoptosis in Amyloid β-Injected Rats Via Inhibition of Nitric Oxide Synthase: Possible Participation of Autophagy

**Published:** 2015

**Authors:** Marjan Shariatpanahi, Fariba Khodagholi, Ghorbangol Ashabi, Azar Aghazadeh Khasraghi, Leila Azimi, Mohammad Abdollahi, Mohammad Hossein Ghahremani, Seyed Nasser Ostad, Farshid Noorbakhsh, Mohammad Sharifzadeh

**Affiliations:** a *Department of Toxicology and Pharmacology, Pharmaceutical Sciences Research Center, Faculty of Pharmacy, Tehran University of Medical Sciences, Tehran, Iran.*; b *Neuroscience Research Center, Shahid Beheshti University of Medical Sciences, Tehran, Iran.*; c *Neuro Biology Research Center, Shahid Beheshti University of Medical Sciences, Tehran, Iran.*; d *Department of Physiology, School of Medicine, Physiology Research center, Ahvaz Jundishapour University of Medical Sciences, Ahvaz, Iran.*; e *Tehran University of Medical Sciences, International Campus, Tehran, Iran.*; f *Department of Neuroscience, School of Advanced Technologies in Medicine, Tehran University of Medical Sciences, Tehran, Iran.*; g *Department of Immunology, Faculty of Medicine, Tehran University of Medical Sciences, Tehran, Iran.*

**Keywords:** MWM, L-NAME, Alzheimer’s disease, Apoptosis, Autophagy

## Abstract

It has been proposed that appearance of amyloid beta (Aβ) in hippocampus is one of the characteristic features of Alzheimer’s disease (AD). The role of Nitric oxide (NO) in neurodegenerative disorders is controversy in different contexts. Here, we examined the effect of NO on spatial memory. For this purpose, we compared the effects of three different concentrations of L-NG-Nitroarginine Methyl Ester (L-NAME) as a nitric oxide synthase (NOS) inhibitor. We used Morris water maze (MWM) for evaluation of behavioral alterations. We also assessed the apoptosis and autophagy markers as two possible interfering pathways with NO signaling by western blot method. We found that in Aβ pretreated rats, intra-hippocampal injection of 1or 2 (μg/side) of L-NAME caused a significant reduction in escape latency and traveled distance comparing to Aβ-treatment group. Our molecular findings revealed that L-NAME could induce autophagy and attenuate apoptosis dose dependently. The protective role of autophagy and the deteriorative role of apoptosis is the hypothesis that can vindicate our findings. Thus using NOS inhibitors at low concentrations can be one of the therapeutic approaches in the future studies.

## Introduction

Recent statistics researches point out that aged human population and related elderly diseases are growing up. Alzheimer’s disease (AD) is the most common age-associated dementia which accounts for an estimated 60 to 80 percent of cases (1). In AD, appearance of amyloid beta (Aβ) in hippocampus is one of the characteristic features of dementia and memory impairment. Despite broad research funding in the field of AD, shortage of knowledge in AD care still exists. Nitric oxide synthase (NOS) can engender NO using arginine as amino acid. NO acts as a neurotransmitter which modulates long-term potentiation and long-term depression and also produces oxidants in the brain (2). Increasing of NO level leads to mitochondrial mediated apoptosis in the neurons against oxidative stress (3). On the other hand, inhibition of NO by L-NG-Nitroarginine Methyl Ester (L-NAME), a general inhibitor of NOS, in the Aβ pretreated rodents and cell cultures attenuates glutamate level and protects cells from apoptosis (4, 5). Moreover, an investigation indicated that NOS inhibitors improved memory impairment in arsenic induced rats (6). So, the dual role of NO in the neuronal system and neurodegenerative diseases is in the conflict by researchers. Autophagy is an intracellular process which eliminates damaged organelles and clears pathogenic long-lived proteins in the cytoplasm. On the other hand, autophgy can be introduced as a kind of cell death. This dual role of autophagy in different situations is on the researchers’ focus (7). There are three kinds of autphagy; macro-autophagy, micro-autophagy and chaperon mediated autophagy. In the current study, we focused on macro-autophagy (hereafter named autophagy) which plays the most significant roles among others (8). LC3 (Light Chain 3) protein is one of the important factors which can trigger autophgay in the cell. Further, autophagy involved proteins, which named “Autophagy Related Genes” (ATGs) can bring on this phenomenon in all eukaryote and prokaryote cells. Protective role of autophagy against apoptosis has been shown in *in-vivo* and *in-vitro *AD models. (9,10). It has been proposed that NO could modulate autophagy in the cells (11). Recent studies demonstrated that inhibition of NOS could increase autophagic factors and thereby warranty cell survival. NO reduces LC3 and beclin-1 levels in neurodegenerative disease models such as Huntington’s disease (HD) and hypoxia model (12). NOS activation induces JNK and AMPK pathway which can subsequently suppress autophagy and trigger cell death in the neurons (13, 14). Besides, the precise molecular mechanisms which can activate autophagy through NO signaling are unknown. In the current study, we determined the exact effect of NOS inhibition by L-NAME, in behavioral spatial learning and memory, neuronal apoptosis and autophagy. Here, we aimed the protective role of autophagy that could defend cell death and improve memory function via NO signaling pathway in Aβ-injected rats.

## Experimental

*Chemicals*

L-NAME (L-NG-Nitroarginine Methyl Ester) was supplied from Sigma (St. Louis, MO, USA). Aβ (1–42) was provided from Genscript (Piscataway, NJ, USA). Ketamine (Alfasan, Holland) and Xylazine (Pantex Holland B.V.) required for anesthesia before surgery. Antibodies aimed for apoptotic markers (Caspase-3, Bcl-2, Bax) and autophagic markers (LC3, ATG7, Beclin 1) and also β-actin were purchased from Cell Signaling Technology (Beverly, MA, USA). Electrochemiluminescence (ECL) kit was obtained from Amersham Bioscience (Piscataway, NJ, USA). Other chemicals which used in this experiment were obtained from commercial sources.

*Animals*

Adult male Wistar rats (200-250 g) obtained from faculty of pharmacy, Tehran University of Medical Sciences, were used in our study. Rats were stored in groups of four in cages. We randomly used them in various treatment groups. The rats kept at the room temperature (25 ± 2 ˚C) under 12 h light–12 h dark cycles. Water and nutriment were freely ready for use. All experiments were done under the standard guidelines of Ethical Committee for the use and care of laboratory animals at Tehran University of Medical Sciences (No. 8069, July 2008).

*Surgery*

First, rats were anesthetized with *i.p* injection of 100 mg/Kg ketamine and 25 mg/Kg xylazine, and then were put in stereotaxic instrument (Stoelting, Wood Dale, IL, USA). Guide cannulas were placed in dorsal hippocampus (CA1) and fixed to the skull surface by orthopedic cement. Cannula placement was performed according to the atlas of Paxinos and Watson (15). (Anterior–posterior, 3.8 mm; media-lateral, ±2.2 mm; dorsa-ventral, 2.7 mm from bregma). Guide cannulas (23 G) and injection needles (30 G) were used for intra-hippocampal infusion. The injection needle was linked to Hamilton syringe by a polyethylene tube. Drug infusion was performed in about one minute. Further sixty seconds was necessary for perfect exit of the drug from the needle tip by keeping the injection needle in place.

*Drug preparations and infusions*

L-NAME was dissolved in Saline (0.9%) to reach the final concentrations of 0.5, 1 and 2 (μg/μL). Aβ was dissolved in PBS and the solution of Aβ was incubated for 5 days at 37 ^◦^C. Aβ was reached to the final concentration of 30 (ng/μL). 1 μL of L-NAME or Aβ were injected in both sides of hippocampus according to mentioned doses. On the surgery day, in treatment groups, 30 min after the infusion of Aβ, L-NAME (0.5, 1 or 2 μg/side) was injected.

*Drug treatment*

 In the present study, animals were randomly divided into eight groups and each group (n = 6) received intra-hippocampal infusions of drugs bilaterally (1 μL/side): (I) Control group receiving normal saline (0.9%); (II) Aβ-treatment group, which received Aβ (30 ng/μL/side) without receiving any treatment; (III-V) L-NAME treatment groups, which received different concentrations of L-NAME (0.5,1 and 2 μg/μL/side); (VI-VIII) groups which received Aβ (30 ng/μL/side) plus mentioned concentrations of L-NAME. The latter groups went through two experimental procedures: behavioral experiments and molecular studies. 

*Behavioral procedure*

Fourteen days after drug injections, behavioral test was performed by Morris water maze (MWM). MWM task and especial considerations in this test have been explained completely (16). A hidden platform was placed in the target quadrant and rats were trained for finding this platform for 4 succeeding days (17). In the image of arena, it had 4 quadrants and the quadrant which the platform was inserted was known as target quadrant. Four trials in a day from different starting places were performed. On the 5th day, the hidden platform was removed and a probe testing trial was carried out. The animal was released from one point and allowed to swim in the pool. The time which the animal spent in the target quadrant was measured for consolidation assessment in treated rats. The rat location and different movement tracks were recorded by a camera above the MWM pool. The recorded tracks were converted to an excel data for evaluating spatial memory through different parameters like escape latency (time spent to find the hidden platform), traveled distance (the length of swimming path to reach the hidden platform), swimming speed and time spent in target quadrant by using EthoVision 3.1 tracking system (Noldus Information Technology, Wageningen, the Netherlands) (18). In our experiment, escape latency, traveled distance and swimming speed as well as time spent in the target quadrant were recorded during a period of 90 s, in probe and training trials. All of the experiments were performed between 8 AM to12 PM.

*Western blot analysis*

Western blot analysis was performed seven days after the drug injections. At first, the homogenization of the extracted hippocampus was performed by using a lysis buffer. Next, the samples were centrifuged at 3000 rpm and 4 °C for five minutes, and the supernatant was separated. The concentrations of the proteins were measured by Bradford’s method (19). Loading buffer was added to the supernatants and heated for 5 minutes. The total proteins were loaded (60 μg protein) on 12% Sodium Dodecyl Sulfate-Poly Acrylamide Gel Electrophoresis (SDS-PAGE).Then; separated proteins were transferred to a polyvinylidenefluride membrane and probed with suitable purpose antibodies. ECL reagents and radiography on BioMax film for detection of immunoreactive polypeptides were used. We used densitometric scan of films by using image J 1.410 (NIH, USA) and normalized to *β*-actin. 

*Statistical analysis*

Results are described as mean ± S.E.M. We used One-Way ANOVA followed by Newman–Keuls multiple comparison post hoc test for analyzing behavioral parameters and Tukey’s multiple comparison post hoc test for comparing molecular scores. We also used two-way ANOVA for comparing the influence of treatment and training days on traveled distance and escape latency as measured factors in each day. p-values less than 0.05 were statistically regarded as significant.

## Results

*Behavioral results*

*Effect of training on escape latency, traveled distance, and swimming speed in the MWM during four testing trials in L-NAME and amyloid beta pretreated animals*

**Figure 1:**
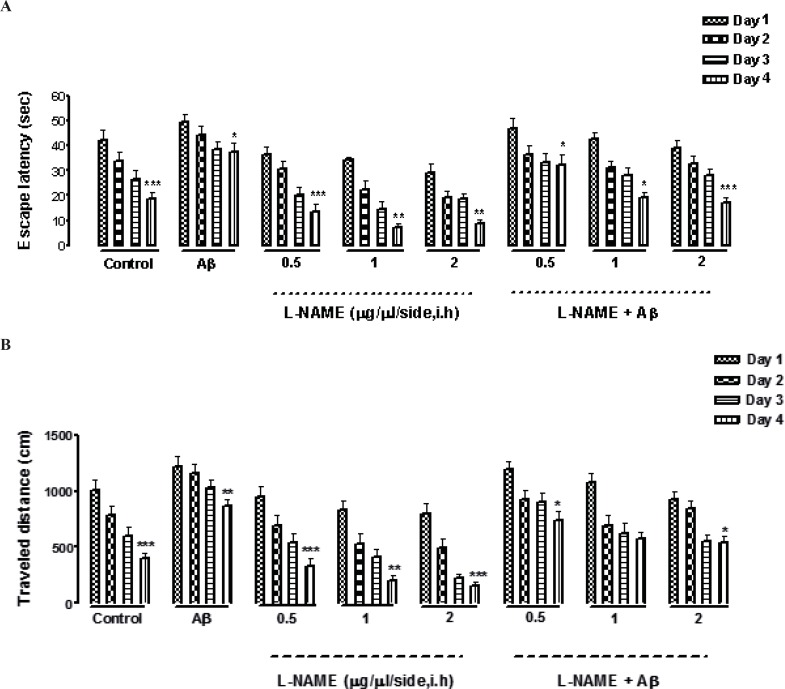
Effects of four-training trials on escape latency (A) and traveled distance (B) in L-NAME and Aβ pretreated animals. There was a significant reduction in escape latency and traveled distance in all the groups through four days of training (Figures 1 A and B). There was a significant difference between the first and the fourth day of training, measured by two factors of escape latency and traveled distance except in (Aβ + L-NAME 1 μg/side) treatment group which the traveled distance didn’t decrease significantly from first day to the fourth day of training. The swimming speed was consistent during the four training days in all of the groups (data not shown). Each value explains Mean ± S.E.M for 6-8 rats. (*P < 0.05; **P < 0.01; ***P < 0.001 shows difference between the first and the fourth days of training).

Escape latency, traveled distance, and swimming speed were three variables which measured in our experiment. Our data shows that all animals including the control group and the groups received different concentrations of L-NAME with or without Aβ learned how to find the hidden platform in the MWM. It was confirmed by finding a consequential reduction in escape latency and traveled distance in all the groups through four days of training (Figures 1 A and B). In addition by comparing the same training days of different groups by two way ANOVA, we found that there was a significant difference between the first and the fourth day of training, measuring by two factors of escape latency (F [3, 7] = 0.5928, P < 0.001, n=8) and traveled distance (F [3, 7] = 0.6482, P < 0.001, n=8) except in one treatment group which received (Aβ + L-NAME1 μg/side). In this group, there was not a significant difference in traveled distance between the first and the fourth day of training. The swimming speed was the same during the training days in all the groups (data not shown). 

*Interactive effect of administration of bilateral intra-hippocampal infusions of Aβ and different doses of L-NAME on escape latency, traveled distance, and swimming speed during training days*

**Figure 2:**
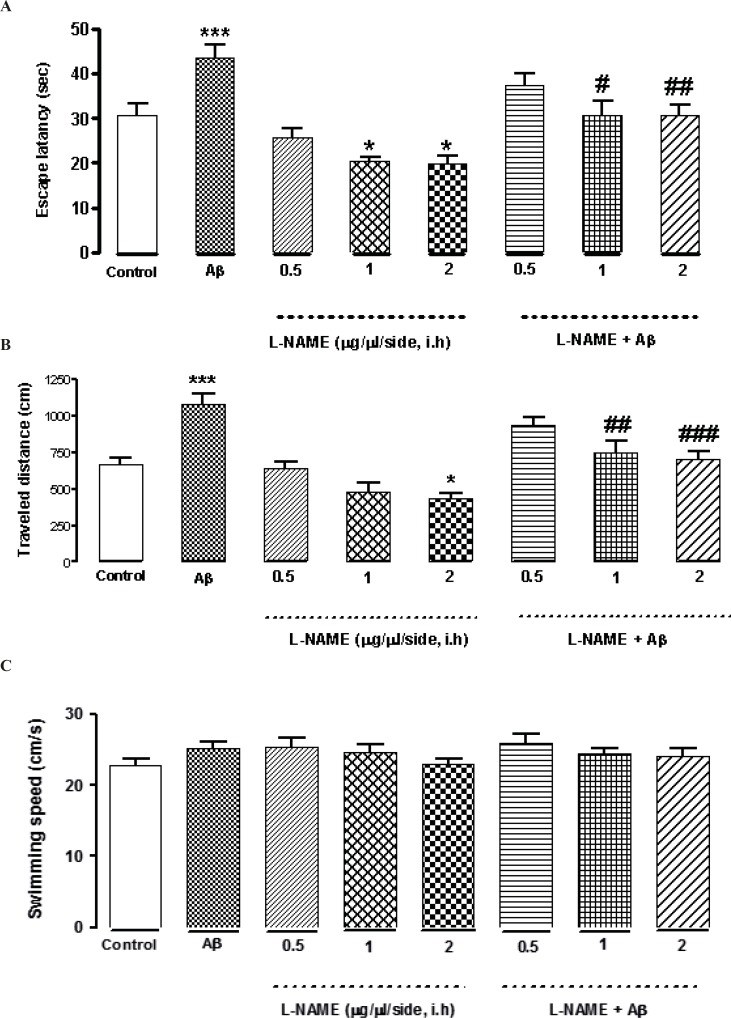
Effects of different concentrations of L-NAME infusion on escape latency (A), traveled distance (B), and swimming speed (C) during the testing trials. Hippocampus infusion of L-NAME (1, 2 μg/side, i.h) showed a significant decrease in escape latency compared to the control group. Treatment by L-NAME (2 μg/side, i.h) decreased traveled distance significantly comparing to the control group (Figures 2 A and B).There was no significant difference in swimming speed between the control and the treatment groups (Figure 2C). Intra-hippocampal infusion of Aβ significantly increased the time and distance for finding the hidden platform comparing with the control group. Intra-hippocampal infusion of 1, 2 (μg/side) of L-NAME with Aβ decreased the time and distance for finding the hidden platform significantly in comparison with Aβ treated animals. Each point explains Mean ± S.E.M for 6-8 rats. (*P < 0.05; ***P < 0.001 different from control group, #P < 0.05; ##P < 0.01; ###P < 0.001 different from Aβ-treated group

L-NAME at concentrations of (0.5, 1and 2 μg/side) was infused bilaterally into the CA1 region of the hippocampus. As shown in Figures 2A and B, intra-hippocampal infusion of L-NAME (1or 2 μg/side) showed a significant decrease in escape latency (*P < 0.05) compared to the control group. Moreover, treatment by 2 (μg/side) of L-NAME, decreased traveled distance significantly comparing to the control group (*P < 0.05).There was no significant difference in swimming speed between the control and the treatment groups (Figure 2C). The results suggest that intra-hippocampal infusion of L-NAME (1or 2 μg/side) improved spatial memory in rats without any effect on motor function, while administration of 0.5 (μg/side) of L-NAME had no effect on spatial memory. On the other hand, bilateral infusions of Aβ (30 ng/side) into the CA1 region of the hippocampus, significantly increased the time and distance (***P < 0.001) for finding the hidden platform comparing with the control group. This data confirms that intra-hippocampal infusion of Aβ impaired spatial memory in the MWM (Figures 2 A and B). Interestingly, administration of 1or 2 (μg/side) of L-NAME with Aβ decreased the time for finding the hidden platform significantly comparing with Aβ-treated animals. (#P < 0.05 by infusion of 1 μg/side of L-NAME and ##P < 0.01 by infusion of 2 μg/side of L-NAME). Also the distance which these two treatment animals swam for finding the hidden platform, decreased significantly comparing with Aβ-treated animals. (##P < 0.01 by infusion of 1 μg/side of L-NAME, ###P < 0.001 by infusion of 2 μg/side of L-NAME). According to our results, there was not a significant difference in escape latency and traveled distance between the latter treatment groups and the control group. It means that infusion of L-NAME (1 or 2 μg/side) could reverse the Aβ-induced memory deficiency. But infusion of 0.5 (μg/side) of L-NAME had not such effect.

*Effect of bilateral intra-hippocampal infusion of Aβ and different doses of L-NAME on time spent in the target quadrant in 90 s probe test*

**Figure 3:**
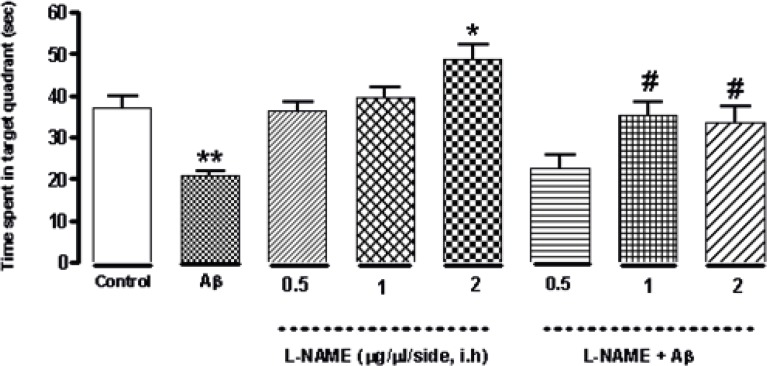
Interactive effect of administration of bilateral intra-hippocampal infusion of Aβ and different doses of L-NAME on time spent in target quadrant in 90 sec probe test. Treatment with L-NAME (2 μg/side, i.h) significantly increased the time animals spent in the target quadrant comparing to control. Furthermore administration of Aβ (30 ng/side) into the CA1 region of the hippocampus decreased this time significantly compared to the control. The time increased significantly by administration of 1or 2 (μg/side) of L-NAME after infusion of amyloid beta comparing to Aβ-treatment group. Each point shows the mean ± SEM for 6–8 rats. (*P < 0.05; **P < 0.01 different from control group, #P < 0.05 different from Aβ-treatment group).

As shown in Figure 3 treatment with L-NAME (2 μg/side, i.h ) significantly increased the time animals spent in the target quadrant in probe test (*P < 0.05) comparing to control group. But administration of 0.5 or 1 (μg/side) of L-NAME did not change this time significantly comparing to the control. Furthermore administration of amyloid beta (30 ng/side) into the CA1 region of the hippocampus decreased this time significantly (**P < 0.01) compared to the control group. The time increased significantly by administration of 1or 2 (μg/side) of L-NAME after infusion of amyloid beta (#P < 0.05) comparing to Aβ-treatment group. Under our investigation, there is not a significant difference in time spent in the target quadrant on the test day between the latter treatment groups and the control group .It means that infusion of 1 or 2 (μg/side) of L-NAME could reverse the Aβ-induced memory deficiency while 0.5 (μg/side) of L-NAME infusion had no effect.

*Molecular results*

*Interactive effect of administration of bilateral intra-hippocampal infusions of Aβ and different doses of L-NAME on expression of Caspase-3, Bcl-2 and Bax as apoptotic markers*

**Figure 4:**
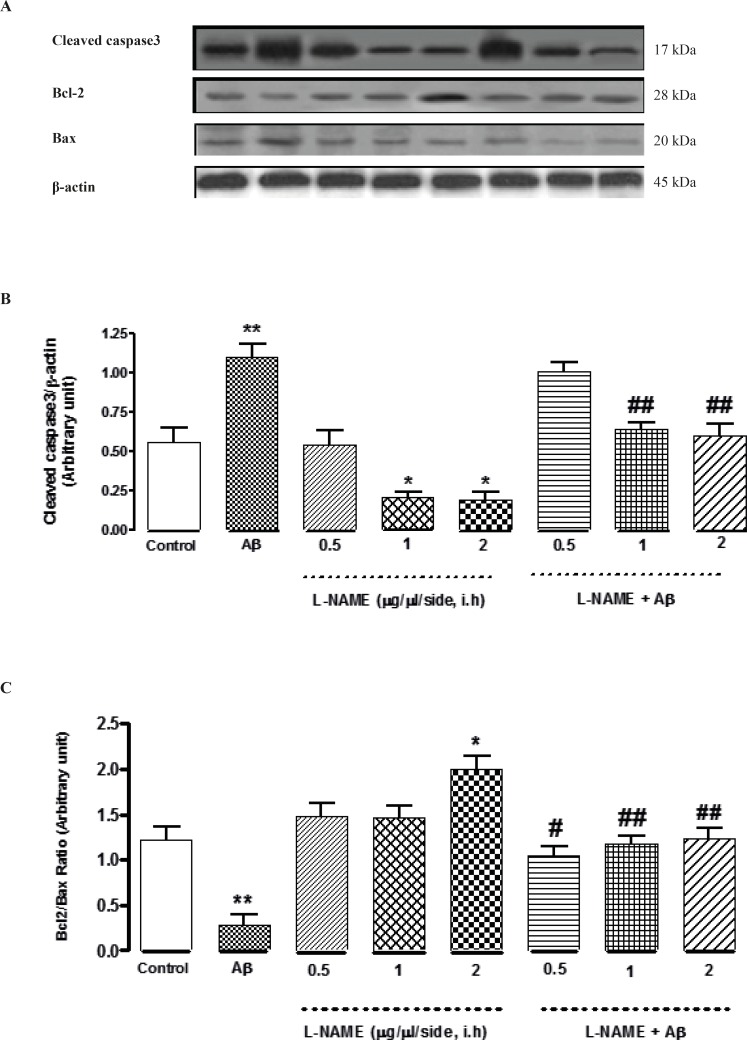
Western blot analysis to measure the effect of L-NAME treatment on the expression of Caspase-3, Bcl-2 and Bax in the hippocampus of rats. (A) 60 μg proteins were alienated on SDS-PAGE, western blotted, probed with specific primary antibodies, and reprobed with control loading antibody (One typical western blot of each antibody is shown: n = 6). (B) The densities of cleaved caspase-3 bands were evaluated and their ratios to β-actin were measured. (C) The densities of Bcl-2 and Bax bands were measured and the ratio of Bcl-2 to Bax was evaluated. Each point shows the mean ± SEM. (*P < 0.05; **P < 0.01 different from the control group, #P < 0.05; ##P < 0.01 different from the Aβ-injected group

To determine whether L-NAME affects apoptosis pathway, we evaluated the level of three important apoptotic markers (Cleaved caspase-3, Bcl-2 and Bax) by Western blot analysis. As shown in Figure 4B, the cleavage of caspase-3 significantly decreased (*P < 0.05) when L-NAME (1 or 2 μg/side) is used comparing to the control group. Densitometric analysis revealed that administration of 1or 2 (μg/side) of L-NAME decreased the level of cleaved caspase-3 about 2.7-fold compared to the control group while administration of 0.5 (μg/side) of L-NAME had no effect on cleavage of caspase-3. In this study, we determined L-NAME’s effect on Bcl-2/Bax ratio too. We found that administration of 2 (μg/side) of L-NAME led to a significant decrease in Bax expression and an increase in Bcl-2 expression comparing to the control group (*P < 0.05). In treatment group with L-NAME (2 μg/side), the ratio of Bcl-2/Bax increased by about 1.6-fold compared to the control group. But administration of 0.5 or 1 (μg/side) of L-NAME did not change the Bcl-2/Bax ratio significantly comparing to the control group (Figure 4C). On the other hand, treatment by Aβ showed a significant increase in cleavage of caspase-3 comparing to the control group (**P < 0. 01). This increase was remarkably attenuated by administration of 1or 2 (μg/side) of L-NAME. Densitometric analysis show that administration of 1or 2 (μg/side) of L-NAME decreased cleavage of caspase-3 by about 1.8-fold comparing to Aβ treatment group (##P < 0.01), While administration of 0.5 (μg/side) of L-NAME had not such effect (Figure 4B). Furthermore, we found that the increase of apoptosis in Aβ-injected rats went along with a significant increase in Bax expression and a decrease in Bcl-2 expression (**P < 0.01). Our results show that in Aβ treatment groups, infusion of 0.5 (μg/side) of L-NAME increased Bcl-2/Bax ratio by 3. 9-fold (#P < 0.05), while administration of 1or 2 (μg/side) of L-NAME increased Bcl-2/Bax ratio by about 4.3-fold (##P < 0.01) comparing to Aβ treatment group. (Figure 4C). Our data provide compelling evidence that there is not a significant difference in expression of apoptotic markers (Caspase-3 and Bcl-2/Bax) between the latter treatment groups (Aβ + L-NAME 0.5, 1 or 2 μg/side) and the control group; it means that L-NAME at mentioned concentrations could significantly reverse the apoptosis caused by Aβ infusion.

*Interactive effect of administration of bilateral intra-hippocampal infusions of Aβ and different doses of L-NAME on expression of LC3, ATG7 and Beclin 1 as autophagic markers*

**Figure 5:**
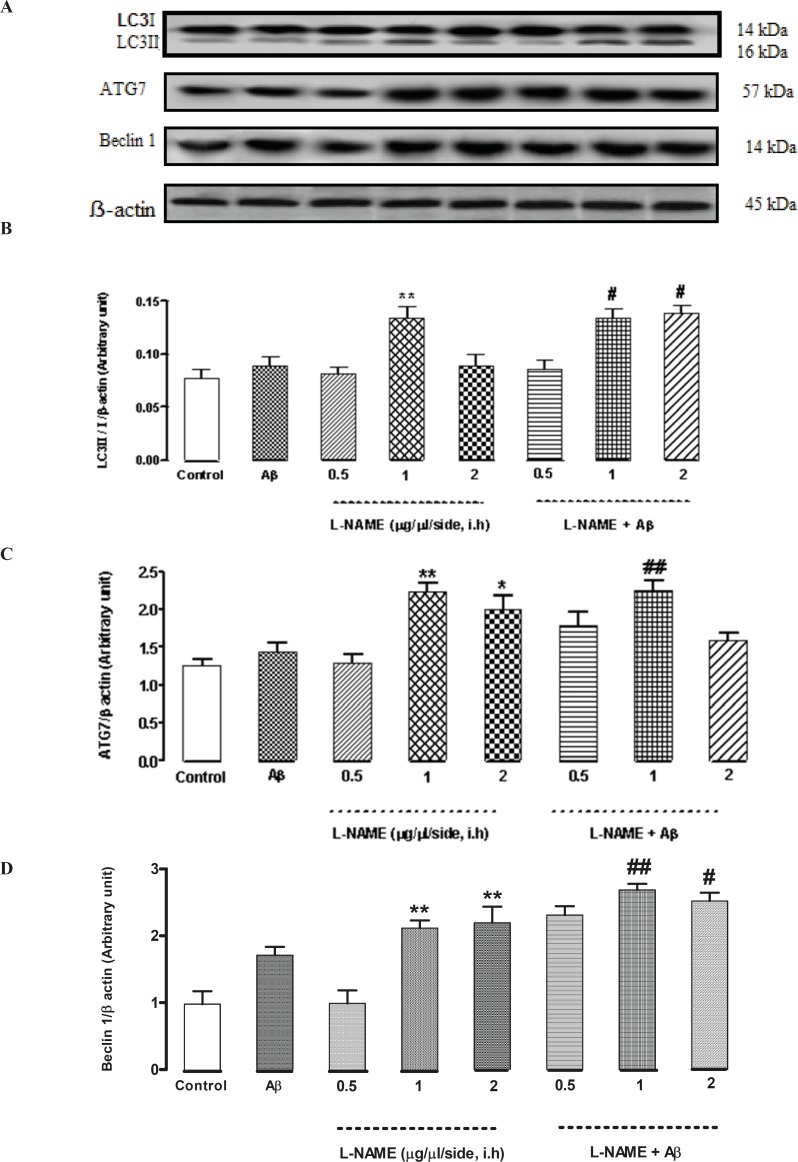
Western blot analysis to measure the effect of L-NAME treatment on the LC3, ATG7 and Beclin 1 expression in the hippocampus of rats. (A) 60 μg proteins were alienated on SDS-PAGE, western blotted, probed with specific primary antibodies, and reprobed with control loading antibody (One typical western blot of each antibody is shown: n = 6). (B) The densities of LC3І and LC3ІІ bands were evaluated and the LC3ІІ/LC3І ratio was measured. (C) The densities of ATG7 bands were evaluated and their ratios to β-actin were measured. (D) The densities of Beclin 1 bands were evaluated and their ratios to β-actin were measured. Each point shows the mean ± SEM. (*P < 0.05; **P < 0.01 different from the control group, #P < 0.05; ##P < 0.01 different from the Aβ-injected group

 To determine whether L-NAME affects autophagy pathway, we evaluated the level of three pivotal autophagic markers (LC3, ATG7, and Beclin 1) by Western blot method. As shown in Figure 5B, LC3II/LC3I ratio significantly increased when 1 (μg/side) of L-NAME is used compared to the control group (**P < 0.01). Densitometric analysis revealed that LC3ІІ/LC3І ratio increased by about 1.7-fold with administration of L-NAME (1 μg/side) comparing to the control group while administration of 0.5 or 2 (μg/side) of L-NAME had no effect on LC3II/LC3I ratio. In this study, we determined L-NAME’s effect on ATG7 protein expression too. The level of ATG7 significantly increased in groups which received 1 or 2 (μg/side) of L-NAME compared to the control group (**P < 0.01 by infusion of 1 μg/side of L-NAME and *P < 0.05 by infusion of 2 μg/side of L-NAME). But administration of 0.5 (μg/side) of L-NAME did not change ATG7 protein level significantly compared to the control group. Densitometric analysis revealed that ATG7 protein level increased by about 2.2-fold with administration of 1 (μg/side) of L-NAME and by about 1.8-fold with administration of 2 (μg/side) of L-NAME comparing to the control group (Figure 5C). Besides, as shown in Figure 5D, treatment with L-NAME (1 or 2 μg/side) led to a significant increase in Beclin 1 protein level compared to the control group (**P < 0.01). Our data shows that Beclin 1 protein level increased by about 2.1-fold in the L-NAME (1or 2 μg/side) treated groups compared to the control group while administration of 0.5 (μg/side) of L-NAME had no effect on Beclin 1 protein expression. On the other hand, L-NAME increased LC3, ATG7 and Beclin 1 levels as autophagic markers in Aβ-injected rats. Treatment by Aβ didn’t show any significant change in the LC3II/LC3I ratio comparing to the control group. In Aβ-treated rats administration of 1or 2 (μg/side) of L-NAME significantly increased LC3II/LC3I ratio by about 1.5-fold in comparison with Aβ-injected rats (#P < 0.05) while administration of 0.5 (μg/side) of L-NAME had not such effect (Figure 5B). Also treatment by Aβ didn’t show any significant change in the expression of ATG7 protein, comparing to the control group. Our data shows that administration of 1 (μg/side) of L-NAME in Aβ-pretreated rats, significantly increased ATG7 protein expression by about 1.5-fold comparing with Aβ-injected rats (##P < 0.01) while administration of 0.5 or 2 (μg/side) of L-NAME had not such effect in Aβ-pretreated rats (Figure 5C). Additionally, we measured the level of Beclin 1 by Western blot analysis. Pretreatment by Aβ didn’t show any significant change in the expression of Beclin 1 protein level comparing to the control group. But administration of 1or 2 (μg/side) of L-NAME in Aβ-pretreated rats, significantly increased Beclin 1 protein expression by about 1.5-fold comparing to Aβ-injected rats (##P < 0.01 by infusion of 1 μg/side of L-NAME, #P < 0.05 by infusion of 2 μg/side of L-NAME), while administration of 0.5 (μg/side) of L-NAME, did not change the Beclin 1 protein expression significantly in Aβ-injected rats (Figure 5D).

## Discussion

An important problem of neurological diseases such as AD, hypoxia, and ischemia, is cognitive dysfunction, that there is a prompt need to focus on the mechanisms of such disorders. In this study we examined NO as the probable interfering factor in AD. We compared the effects of different concentrations of L-NAME as a NOS inhibitor by testing behavioral and molecular alterations. As it is proved in other studies, NO makes soluble guanylate cyclase which will produce cGMP. Production of cGMP can lead to activation of PKG in the neurons (20, 21). It is confirmed that NO has an important role in physiology and pathology of AD (22, 23). Recent studies show that NO is a major molecule that regulates some physiological process of neurons in brain (24-26). All of the studies showed that NOSs have an important role in neurodegenerative disorders in rodents. The best way to understand the biological mechanism of NO is using different concentrations of NO (27). It has been shown that the rats which were infused by Aβ, had high nNOS or iNOS activity (28). Studies showed that the synthesis of NO and nitration of proteins could impair cognitive behavior. In brain seizures which caused by Aβ (25–35) peptide, iNOS has an important role, because it can produce excessive NO which can lead to cognitive deficiency (29). Moreover, there are many antithetic studies about the role of NO in neurodegenerative diseases. It has been shown that nitrite level went up in Aβ (25–35)-treated group and L-NAME pretreatment could reverse these changes (30). Concentration of NO is important in its reaction under cellular stress. At low concentrations of NO, oxidation of the substrate is predictable, on the other hand at high concentrations, NO has nitrosative effect that leads to nitration of proteins and neuronal death (29). It means that there is a U-shaped dose-effect curve. When drugs are affecting through various mechanisms and biological systems we will have such U-shaped curves .There are some hypothesis about these curves such as tachyphylaxis or receptor fatigue that leads to asymptotic response in high doses of drugs (31). Some authors claim that the distinct effects of low concentrations of NO are linked to inhibition of caspases and apoptosis which cause memory improvement. Besides, by oxidative stress induced by amyloid beta, the nNOS activity may be decreased and subsequently iNOS activity will be increased which can cause memory impairment and toxicity (29). Although the role of NO in brain is complex and has controversy but it is proved that high doses of L-NAME can impair memory while low doses of L-NAME can promote memory and these low doses of L-NAME, can antagonize memory deficiencies that caused by decreasing of NMDA, as there are investigations showing that using a NMDA receptor antagonist can lead to memory impairment (32-34). An investigation confirmed that reduction in NO level has a protective role in HD by interfering different pathways because one reason of toxicity is activation of NMDA receptors that leads to neurotoxicity in neurodegenerative disorders like HD, PD and AD and another reason would be overproduction of NO that can cause excitotoxicity (35). We can conclude that time of administration and concentration of NO are two predominant factors in NO function (27, 32). Moreover, our study indicated that treatment of animals by distinct concentrations of L-NAME could decrease escape latency, traveled distance and apoptotic proteins levels compared to control group. Also according to our behavioral and molecular results, L-NAME could reverse the Aβ-induced memory deficiency by decreasing the apoptotic markers in a dose dependent manner. It is important to note that behavioral and molecular evaluations were determined in two different set points. Molecular changes debuts faster and earlier than behavioral changes (36). Molecular alterations have occurred within 7 days but they have decreased at 20^th^ day, while the behavioral changes were detectable by MWM test. It shows that molecular changes occur before the onset of behavioral abnormalities in Aβ-injected rats (37). According to mentioned papers, high doses of NO can activate apoptosis and our results point out that tiny inhibition of NOS protects brain against Aβ related toxicity. Our data in this study indicate the involvement of NO in AD experimental model. On the other hand, we evaluated autophagy markers to determine its probable interference with NO signaling in Aβ-treated rats. Neurodegenerative disorders may produce mutant proteins that can be removed with autophagy through degradation and also any problem in autophagy pathway will cause accumulation of their toxicity (38, 39). Investigations show that NO can diminish autophagy process by preventing from autophagosome formation. Interestingly, NO can reduce autophagy in different cell lines by two main mechanisms: 1. NO prevents the activity of Jun N-terminal kinase1 (JNK1) by S-nitrosylation that leads to reduction in Bcl-2 phosphorylation. So Bcl-2–Beclin 1 interaction will be increased and Beclin 1–hVps34 association will be disturbed. 2. NO can activate mTORC1 by interfering of TSC2 and IKKβ. These two mechanisms are not dependent to each other and are active in autophagy caused by starvation (12). On the other hand, some researches claim that NO activates autophagy related genes in the cells (40). Other investigations confirm that autophagy inducers can be protective in neurodegenerative disorders (39, 41). The paradoxical role of autophagy in the brain still remains unclear. Our results show that the protective concentrations of L-NAME can enhance autophagy pathway comparing to the control group. Conceivably, autophagy activation in rats treated by effective concentrations of L-NAME, increased cell survival and improved memory in Aβ-pretreated rats. So, we suggest that function of autophagy can be protective in the current study. A recent time dependent study showed that after intra-hippocampal infusion of Aβ, autophagy process became active, whereas the autophagic markers increased till the 7^th^ day after Aβ injection and afterwards, the autophagy markers decreased in a temperate manner. On the other hand, apoptosis pathway, started to increase in Aβ-treated rats significantly on 7^th^ day after its injection (42). Here, in our study we can justify our results in the same way. It means that on 7^th^ day after Aβ injection, we evaluated the molecular changes and we found an increase in apoptotic markers while autophagy markers did not increase significantly. Besides, using L-NAME at distinct concentrations could attenuate the Aβ-induced apoptosis and also enhance autophagy significantly. We can conclude that reduction in autophagy may lead the degraded neurons to apoptotic pathway (43).

## Conclusion

In conclusion, inhibition of NOS enhances spatial memory consolidation and attenuates apoptosis dose dependently. Our results suggest that activation of autophagy by a NOS inhibitor can lay off progress of neurodegeneration in AD rat models. The protective role of autophagy and the deteriorative role of apoptosis is the hypothesis that can be dependent to various factors, so some more investigations (using autophagy inducers or inhibitors in AD rats with or without L-NAME) might be performed to elucidate the exact role of autophagy in NO signaling. 
